# Stimuli‐responsive nanostructured hydrogen sulfide donors for enhanced cancer treatments

**DOI:** 10.1002/smo2.70076

**Published:** 2026-06-24

**Authors:** Yuxuan Lyu, Jiaxuan Zhu, Lei Lei, Qingyang Zhong, Zhao Li, Caihong Lei, Fan Rong, Yin Wang

**Affiliations:** ^1^ School of Pharmacy Shihezi University Shihezi China; ^2^ International Joint Laboratory of Green Textile (Zhejiang Sci‐Tech University) Ministry of Education Engineering Research Center of Cell & Therapeutic Antibody Shanghai Frontiers Science Center of Drug Target Identification and Delivery National Key Laboratory of Innovative Immunotherapy School of Pharmaceutical Sciences Shanghai Jiao Tong University Shanghai China; ^3^ Qujing University of Medicine & Health Sciences Qujing China; ^4^ Department of Gastroenterology and Hepatology Zhongshan Hospital Fudan University Shanghai China; ^5^ InnoPep, Inc. San Diego California USA; ^6^ International Joint Laboratory of Green Textile (Zhejiang Sci‐Tech University) Ministry of Education Zhejiang Provincial Key Laboratory of Fiber Materials and Manufacturing Technology Zhejiang Sci‐Tech University Hangzhou China

**Keywords:** cancer treatment, controlled drug delivery, hydrogen sulfide, nanostructures

## Abstract

Cancer has become one of the leading causes to human death, which promotes scientists to develop various treatment modalities. Among them, hydrogen sulfide (H_2_S)‐based gas therapy (GT) emerges as a facile but promising approach due to its unique merits including fast active tissue and cell penetration, robust bioactivity, negligible drug resistance. However, as a potent bioactive gas molecule, uncontrolled diffusion would significantly compromise the therapeutic efficacy and cause side effect. To address this issue, stimuli‐responsive nanostructured H_2_S donors, a subtype of donors capable of forming nanostructures, have been explored. Compared with unassembled ones, they could not only prolong the release time and fine tune the release rate at the aid of nanostructures, but also achieve targeting delivery of H_2_S in a passive or active manner. Given that huge advancement has been made in the past few years, in this review, we survey and categorize the latest progress of these nanostructured H_2_S donors for cancer treatment in terms of different constituted materials from organic to hybrid. Both H_2_S‐based single GT and combined therapies are examined in this review thoroughly. At the end, future outlooks and upcoming challenges of these emerging donors are discussed. We believe that it would inspire scientists to design new donors and extend their applications for human care.

## Graphical abstract

Three typical kinds of stimuli‐responsive nanostructured hydrogen sulfide (H_2_S) donors are reviewed. The nanostructures ensure precise delivery of H_2_S to the lesion site and regulate the release behavior. The H_2_S‐based gas therapy has been used to synergize with other cancer treatment modalities, achieving enhanced efficacy while reducing side effects.

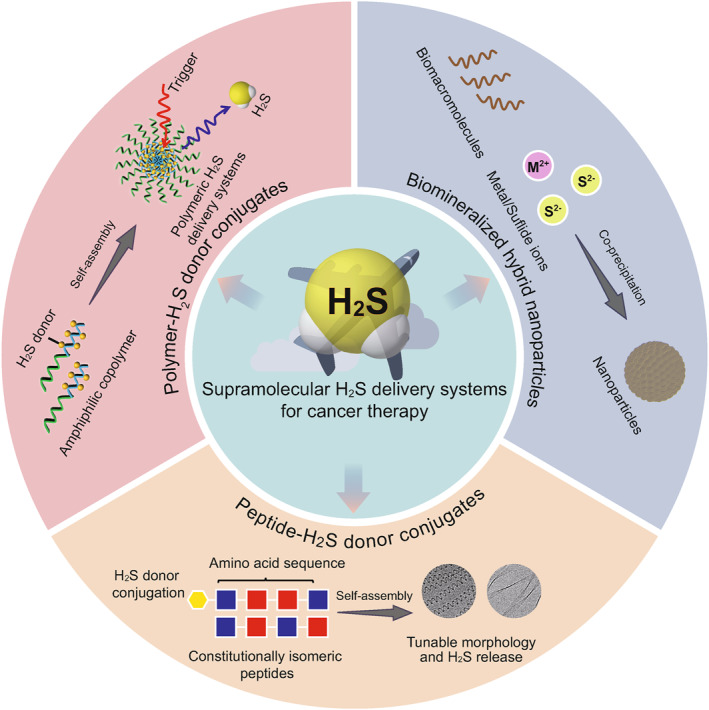

## 1 INTRODUCTION

Cancer has become one of the leading causes to human death.^[1]^ It is primarily treated with surgery, chemotherapy, or radiotherapy. However, surgery cannot completely eradicate cancer cells especially these on the periphery of fragile organs. Meanwhile, frequent administration of chemotherapy increases the possibility of drug resistance formation and induces serious side effects. Those not only compromise the efficacy of these treating modalities, but also reduce patients' life quality and burden the society. To reverse the dilemma, tremendous efforts have been devoted to exploiting new approaches including immunotherapy,chemodynamic therapy (CDT) in the past few years.^[2]^ Among them, gas therapy (GT) receives considerable attention because it, to some extent, outperforms over strategies aforementioned in several aspects.[^3^, ^4^, ^5^, ^6^] On one hand, it employs gas as the drug, which could freely penetrate into the tissues or cells fast and rarely induces drug resistance because gas molecules usually exert their effects on several signaling pathways simultaneously, which makes cells not generate resistance easily. On the other hand, gas molecules especially gasotransmitters participate in many (patho)physiological processes, which somehow manifests GT as a “green” strategy with high biocompatibility and negligible side effects. For instance, hydrogen sulfide (H_2_S) could promote the cell migration and facilitate the wound healing. Interestingly, it is vital for the progression of colorectal cancer and the concentration (0.3–3.4 mM) is much higher in colorectal cancer tissues than in normal counterparts.^[7]^

H_2_S is the last recognized gas molecule joining the gasotransmitter family but gradually gets the same attention as the other two, that is, nitric oxide (NO) and carbon monoxide (CO) in these years.^[8]^ H_2_S is predominantly synthesized from l‐cysteine by cystathionine γ‐lyase (CSE) in the heart and vasculature or cystathionine β‐synthase (CBS) in the brain and nervous system.^[9]^ It can also be generated by nonenzymatic ways, that is, the gut microbiota and by exogenous thiol compounds.^[10]^ Since there are many excellent reviews discussing different functions of H_2_S,[^11^, ^12^], in this review, only the anticancer capacity would be emphasized. Similar to NO and CO, H_2_S exerts its function highly dependent on the concentration (when the concentration is low, it acts protective ability, whereas, high concentration induces toxicity) and cell types. Typically, as a strong reductant, H_2_S could protect normal cells from oxidative stress‐induced injury by directly reacting with reactive oxygen species (ROS) or regulating the inflammation‐related pathways such as NF‐*κ*B to lower the concentration. However, such property is quite different in cancer cells. Unlike protective effect, H_2_S inhibits the catalase (CAT) activity of cancer cells, thereby augmenting the concentration of ROS and inducing strong oxidative stress in situ, which would induce cell apoptosis. Moreover, H_2_S could disrupt the pH homeostasis of tumors by inhibiting anion and sodium/proton exchangers, further to exacerbate intracellular acidosis in cancer cells, which would also induce cell apoptosis. In terms of these, H_2_S has drawn significant interest as a potential therapeutic for cancer treatment. Though direct usage of H_2_S gas is facile for the treatment, H_2_S‐releasing compounds (termed H_2_S donors) are commonly applied instead in these days for the purpose of well controlling the administrated dosage and potentially mitigating side effects.[^13^, ^14^, ^15^] These compounds include inorganic salts [sodium hydrosulfide (NaHS) and sodium sulfide (Na_2_S)],[^16^, ^17^] biomineralized hybrid nanoparticles,^[18]^ combinations of small molecular H_2_S donors with other drugs,[^19^, ^20^, ^21^] small synthetic organic molecules,[^22^, ^23^] peptide‐H_2_S donor conjugates (PHDCs),[^24^, ^25^, ^26^] and polymer/nanoparticle‐H_2_S donor conjugates,[^27^, ^28^, ^29^] among others.

To achieve precise delivery of these H_2_S donors to the lesion site, platforms are usually incorporated especially for those hydrophobic donors. As such, it not only increases the bioavailability of donors and H_2_S, but also decreases the side effects caused by the random diffusion because H_2_S release could only be triggered at the lesion site. Beyond as a reservoir, nanostructures could also modulate the release time/behavior of H_2_S because the packing mode of donors with various structures are usually different. Given these advantages, in this review article, we would like to focus on those nanostructured H_2_S donors capable of responding to specific stimuli, whose H_2_S release behavior could be triggered and tuned by the stimuli. Particularly, we carefully analyze the design rationale respectively in terms of different materials and discuss how H_2_S synergizes other modalities for enhanced therapeutic outcomes because the threshold of GT relied on H_2_S only is typically high. Notably, to demonstrate the latest progress and reflect the design trend, most of examples chosen for discussion were published in recent 5 years. It is our hope that this review could provide some insights into the development of novel nanostructured H_2_S donors and enhance the therapeutic efficacy of H_2_S‐synergized combined therapy for cancer treatments by rational design.

## 2 THE THERAPEUTIC EFFECTS OF H_2_S IN CANCER TREATMENTS

Given that there are several excellent reviews elucidating the role of H_2_S in cancer,[^30^, ^31^] detailed description is omitted here. In brief, H_2_S‐producing enzymes are highly expressed in numerous kinds of cancer cells and are vital for cell survival.^[32]^ It is found that endogenous H_2_S or a relatively low level of exogenous H_2_S could promote proliferation of cancer cells, whereas exposure to a higher concentration of H_2_S or for a long period may result in cancer cell death. In this regard, precise inhibition of H_2_S‐producing enzymes in cancer cells or supplementation of exogenous H_2_S at high concentrations would be beneficial for cancer treatment. The anticancer effect of H_2_S could be achieved via complementary mechanisms, that is, induction of apoptosis, and inhibition of cancer cell proliferation via suppression of DNA synthesis and induction of cell cycle arrest. H_2_S at high concentrations is capable of inhibiting the complex IV, vital for ATP synthesis, thus causing apoptosis.^[33]^ As we know, the cell cycle consists of distinct phases: G1 (pre‐DNA synthesis growth), S (DNA synthesis), G2 (post‐DNA synthesis preparation), and M (mitosis). The proper interaction of various cyclins and cyclin‐dependent kinases (CDKs) ensures the proper functioning of the cell cycle.^[34]^ By persulfidating CDK1 at Cys83, H_2_S disrupts its binding to Cyclin B1, impairing CDK1 kinase activity and arresting cells at G2/M phase. Parallelly, H_2_S stabilizes p53 through persulfidation of MDM2, which suppresses MDM2‐mediated ubiquitination and proteasomal degradation of p53. Then, the accumulated p53 transcriptionally activates p21, a potent CDK inhibitor that inhibits Cyclin E‐CDK2 activity, enforcing S‐phase arrest. Collectively, H_2_S may interact with the p53 protein through various signaling pathways, thereby influencing the cell cycle. Through concomitant CDK inhibition and p53/p21 pathway activation, H_2_S induces reversible cell cycle arrest independent of cell cycle phase. This finding manifests H_2_S as a master redox rheostat that balances proliferation and apoptosis in malignant cells dynamically. It should be noted that precise suppression of these enzymes only in cancer cells is not easy because they are also existing in normal cells as well. Inhibiting the activities would interfere the viability of normal cells and may cause lethal side effects, that is, off‐target toxicity. In this regard, on‐demand delivery of exogenous H_2_S to the lesion site is a facile and promising approach compared with the inhibition one. In terms of the concentration‐dependent bioactivity of H_2_S, precise loading of H_2_S donors into platforms and delivery to the lesion site, followed by the control release is the prerequisite to achieve the satisfactory outcomes.

## 3 NANOSTRUCTURED H_2_S DONORS

### 3.1 Controlled delivery by nanostructured polymer‐H_2_S donor conjugates

**FIGURE 1:**
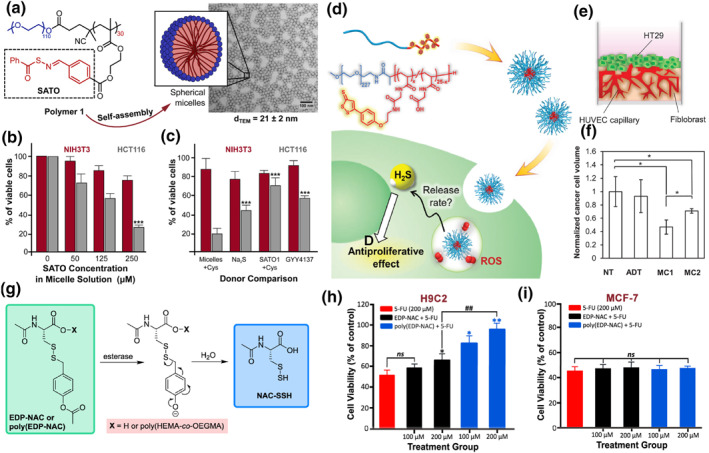
Polymer‐H_2_S donor conjugates for cancer therapy. (a) Formation of SATO‐conjugated polymeric micelles. (b, c) Selective toxicity of SATO‐conjugated micelles to cancer cells. Reproduced with permission.^[35]^ Copyright 2017, ACS. (d) The chemical structure of ADT‐conjugated polymeric micelles. (e, f) Anti‐proliferative effect of ADT‐conjugated micelles against HT29 cancer cells. Reproduced with permission.^[36]^ Copyright 2023, Wiley. (g) The chemical structure of esterase‐triggered persulfide‐prodrug. (h, i) Role of persulfide‐prodrug in mitigating 5‐FU‐induced toxicity selectively. Reproduced with permission.^[37]^ Copyright 2020, ACS.

As discussion above, platforms are commonly used to transport hydrophobic H_2_S donors to the lesion site. There are two ways to load H_2_S donors into the platforms, that is, physical encapsulation or covalent conjugation (the prodrug strategy). Compared with physical encapsulation, covalent conjugation is featured as stable donor loading as the covalent bond between the platform and the donor would not be cleaved until the donor reaches the lesion site if properly designed (Table 1). Importantly, it enables precise donor loading both in the amount and the conjugation site.^[43]^ Inspired by the polymer‐drug conjugate, the Matson research group reported the first example of polymer‐H_2_S donor conjugates (Figure 1a).^[35]^ They appended *S*‐aroylthiooximes (SATOs), a type of cysteine‐responsive hydrophobic donor, to the side chains of poly (ethylene glycol)‐*b*‐poly(2‐(4‐formylbenzoyloxy)ethyl methacrylate)) [PEG‐*b*‐P(FBEMA)] block copolymers. After conjugation, the resultant amphiphilic polymer assembled into spherical micelles in water, which elevated the solubility of SATOs and extended the release half‐time by 6 times. Interestingly, in vitro experiments unraveled that these H_2_S‐releasing micelles exhibited selective toxicity towards colon carcinoma HCT116 when the concentration raised beyond 50 μM (Figure 1b,c). It may be attributed to the H_2_S released from micelles in the presence of cysteine because H_2_S could inhibit the catalase activity of cancer cells and in turn increase the concentration of ROS, ultimately inducing cell death. Similar phenomenon was also found in other cancer cells.^[44]^ Likely, similar approach was also adopted by Hasegawa and coworkers (Figure 1d). They conjugated the H_2_S donor anethole dithiolethione (ADT) to the PEGylated polymer, which formed micelles in water as well due to its amphiphilicity.^[36]^ By changing the numbers of ADT groups in the conjugates, the H_2_S release rate could be finely tuned, thereby impacting the anticancer ability. Both 3D cancer model (Figure 1e,f) and the chick chorioallantoic membrane assay demonstrated that the micelles exhibiting moderately slow H_2_S release exerted a stronger anti‐proliferative effect. This example clearly illustrated that the amount of loaded donors could influence the H_2_S release behavior, which is vital for achieving the anti‐proliferative activity in cancer cells. Notably, though the prodrug strategy ensures the stable conjugation of H_2_S donors to platforms, it would still suffer from dilution‐induced dissociation in physiological conditions if the critical aggregation concentration is high. In order to address this issue, Li and coworkers developed core‐cross‐linked micelles by using di‐*para*‐substituted SATOs (*p*‐diSATOs) as a linker, which integrated cross‐linking of micelle core and conjugation of H_2_S donors through one simple reaction. However, these micelles were not used for anticancer treatment.^[45]^

**TABLE 1:** Summary of nanostructured polymer‐H_2_S donor conjugates.

Donor	Matrix	Trigger	Models	Active molecules	Reference
SATOs	PEG‐*b*‐P(FBEMA)	Cysteine	Colorectal carcinoma HCT116 cells	H_2_S	[35]
Anethole dithiolethione (ADT)	PEGylated polymer	ROS	Human colon cancer HT29 cells, in vitro 3D tumor model,in ovo chick chorioallantic membrane (CAM) tumor	H_2_S	[36]
Ester disulfide‐prodrug *N*‐acetyl cysteine (EDP‐NAC)	poly(HEMA‐*co*‐OEGMA)	Porcine liver esterase	MCF‐7 cells	5‐FU, H_2_S	[37]
Poly(thiocarbamate)	H_2_S‐PTC‐PEG	H_2_S	MCF7/ADR tumor	DOX, H_2_S	[38]
Trisulfide bonds	A trisulfide bond containing polymers (Poly3S)	GSH	4T1 tumor	Pt(IV), H_2_S	[39]
PEG‐DTC	Polyphenolic semiconductor polymer/poloxamer F127	H^+^	4T1 breast cancer	Hafnium, H_2_S	[40]
Trisulfide bonds	IR‐FEP‐RGD‐S‐S‐S‐Fc	GSH	4T1 breast cancer	·OH/heat from HPTT/H_2_S	[41]
2,2′‐dipyridyl tetrasulfide	Conjugated polymer/PEG	GSH	4T1 breast cancer	Heat from PTT, H_2_S	[42]

Different from appending donors to the side chains of polymers, the Matson group reported another polymer‐H_2_S donor conjugate by immobilizing carbonyl sulfide (COS) donors in the main chain of poly(thiourethane) (PTU).^[46]^ PTU was initially synthesized by self‐polyaddition of 4‐isothiocyanatobenzyl alcohol, followed by end capping with (4‐azidophenyl)methanol. In the presence of reductants such as tris(2‐carboxyethyl)phosphine (TCEP), PTU underwent fast depolymerization and concurrently released COS, which then converted into H_2_S by the ubiquitous enzyme carbonic anhydrase (CA). Although it is a smart way to incorporate H_2_S donors in the backbone of polymers, the degree of polymerization is low because of the solubility issue especially when the molecular weight of resultant polymers is high during the polymerization. Besides, it has been proven that persulfide participates in H_2_S signaling pathway.[^11^, ^47^] Compared with H_2_S, it possesses much stronger reduce capacity and enhanced nucleophilicity.^[48]^ Therefore, it is more difficult to transport persulfides to the lesion site. To this end, Matson and coworkers linked the persulfide donor ester disulfide‐prodrug *N*‐acetyl cysteine (EDP‐NAC) to the side chains of the block copolymer poly[2‐hydroxyethyl methacrylate‐*co*‐oligo(ethylene glycol) methacrylate] [poly(HEMA‐*co*‐OEGMA)] through EDC coupling, offering poly(EDP‐NAC) (Figure 1g).^[37]^ Treated with porcine liver esterase, poly(EDP‐NAC) gradually decomposed to release persulfides and mitigated the toxicity induced by 5‐fluorouricil (5‐FU) towards cardiomyocyte H9C2 cells (Figure 1h) while not interfering the chemotherapy (Figure 1I). These results implied that rationally combing reactive sulfide species with chemotherapy could mitigate the side effects on normal tissues while not interfering the efficacy of chemotherapy. It should be noted that all the examples selected in the section focused on the preparation of polymer‐H_2_S donor conjugates and did not discuss the anti‐cancer mechanism of H_2_S. Moreover, almost all the nanostructures are spherical micelles, the influence of different morphologies on the release rate of H_2_S did not carry out.

**FIGURE 2:**
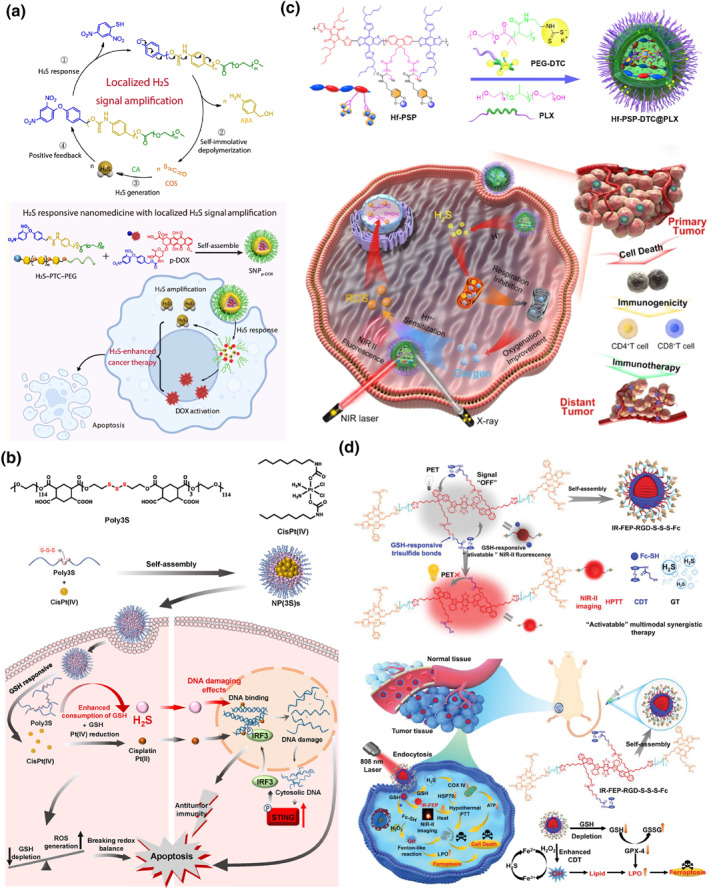
Polymer‐H_2_S donor conjugates for H_2_S‐synergistic cancer therapy. (a) Self‐amplified H_2_S generation of polythiocarbonates and its role to enhance the chemotherapy of DOX. Reproduced with permission.^[38]^ Copyright 2024, Spring Nature. (b) Trisulfide bond containing polymer‐based cisplatin delivery enabling GSH‐activated synergistic therapy. Reproduced with permission.^[39]^ Copyright 2024, ACS. (c) Poly‐dithiocarbamate‐based, semiconducting polymer‐loaded nanosensitizer for H_2_S enhanced RT. Reproduced with permission.^[40]^ Copyright 2022, Wiley. (d) IR‐FEP‐RGD‐S‐S‐S‐Fc nanoplatform for TME‐activated multimodal synergistic therapy. Reproduced with permission.^[41]^ Copyright 2023, Wiley.

Considering that the potency of mono‐GT is not sufficient enough, combination GT with other modalities has become popular to combat cancers. Against the trace amount of H_2_S at the tumor site, Yuan and coworkers developed a 4‐(2,4‐dinitrophenoxy) phenyl) methanol end‐caped H_2_S‐responsive self‐immolative poly(thiocarbamate) for self‐amplified H_2_S imaging and cancer therapy.^[38]^ The amphiphilic diblock polymer H_2_S‐PTC‐PEG could self‐assemble to micelles and meanwhile load H_2_S‐responsive dye or DOX prodrug (Figure 2a). The dye‐loaded nanoprobe could sense the trace amount of H_2_S at the tumor site by localized H_2_S signaling amplification. More importantly, DOX prodrug loaded nanomedicine could overcome the cancer drug resistance due to the energy blockader and substrate efflux pump dysfunction induced by H_2_S through inhibition of mitochondrial COX IV activity and P‐glycoprotein (P‐gp). Consequently, the DOX efflux rate was reduced to 20.85 ± 2.11% whereas this reached 71.62 ± 3.04% when incubated with DOX alone. Besides, Xiao and coworkers prepared reduction‐responsive nanoparticles (NP(3S)s) from a trisulfide bond containing polymers (Poly3S) for the delivery of platinum(IV) (Pt(IV)) prodrug.^[39]^ Upon encountering intracellular glutathione (GSH), both Pt(II) and H_2_S could be liberated from NP(3S)s, leading to potent anticancer effects. Compared with Pt(IV) prodrug, introduction of the trisulfide bond to the polymer increased the consumption of GSH due to the sulfide exchange reaction between trisulfide bonds and GSH, which is almost 2.5 times higher than its counterparts. Therefore, it disrupted the redox balance, and elevated the ROS in situ. Consequently, NP(3S)s were able to induce DNA damage and activate the STING pathway to promote activation of T cells due to more GSH assumption and enhanced Pt accumulation (almost 400 times than CisPt and 4 times than CisPt (IV)). Importantly, such formulation could minimize the liver toxicity caused by cisplatin due to the preferred NP accumulation in tumor (Figure 2b). Collectively, in these examples, H_2_S plays as a booster to augment the therapeutic efficacy while reducing the drug resistance and side effects of chemotherapy.

Radiotherapy (RT) using beams of intense energy to kill cancer cells is another commonly applied method. Compared with chemotherapy, RT offers more precision to the lesion site due to the application of beam. Nevertheless, the efficacy is significantly limited by the hypoxia tumoral environment. To reprogram the oxygen metabolism and elevate the oxygen utilization, Dai and coworkers reported a metalphenolic nanosensitizer (Hf‐PSP‐DTC@PLX) for enhanced RT (Figure 2c).^[40]^ The nanosensitizer was made from an acid‐responsive H_2_S donor polyethylene glycol‐*co*‐polydithiocarbamates (PEG‐DTC) and a hafnium‐chelated polyphenolic semiconducting polymer (Hf‐PSP) wrapped by an amphiphilic polymer (poloxamer F127, PLX). Under acidic tumor microenvironment, Hf‐PSP‐DTC@PLX gradually unleashed H_2_S to inhibit mitochondrial respiration, thereby increasing the percentage of dysfunctional mitochondria from 44.6 % (control group) to 75.1 %, and lowering the metabolic oxygen consumption. Diminished oxygen consumption rate (OCR) was found in cells treated with Hf‐PSP‐DTC@PLX at 1/2 IC50, evidencing the H_2_S‐inhibited cellular respiration with an inhibition ratio of 33 % in basal OCR and 20 % in maximal OCR. In contrast, bare OCR was found with the treatment of Hf‐PSP‐DTC@PLX at IC50 because of the serious cell death. As a result, the radiosensitization effect of Hf‐PSP‐DTC@PLX and ROS production were augmented. Together, the excellent inhibition capability of oxygen consumption and enhanced radiosensitization intensified the RT efficacy with remarkable anti‐tumor immunogenicity. Later, the same research group demonstrated that combination of H_2_S‐based GT with photothermal therapy (PTT) could alleviate the PTT‐induced inflammation because H_2_S can inhibit the NF‐*κ*B pathway and downregulate inducible nitric oxide synthase (iNOS) by sulfide for a suppressed inflammatory stimulation, whereas, it did not attenuate the immune therapeutic performance of PTT (Figure 2d). Therefore, the H_2_S synergized PTT in a single nanoplatform could be used to modulate both inflammatory and adaptive immune response.^[42]^

Besides, Yang and coworkers adopted a similar strategy.^[41]^ They constructed a tumor microenvironment (TME) activatable nanoplatform IR‐FEP‐RGD‐S‐S‐S‐Fc for NIR‐II fluorescence imaging‐guided cancer therapy. At the aid of cRGDfk peptide, IR‐FEP‐RGD‐S‐S‐S‐Fc could home to the tumor site and quickly internalize into cancer cells. By GSH shearing, the trisulfide bonds between the fluorophore and quenched agent were cleaved, leading to tumor site cascade‐specific illumination. Notably, IR‐FEP‐RGD‐S‐S‐S‐Fc acquired CDT/HPTT/GT trimodal synergistic enhancement with laser irradiation, which further benefited the GSH depletion and •OH generation. Besides, the liberated H_2_S amplified oxidative stress and inhibited COX IV activity. The low expression of COX IV diminished the intracellular ATP supply, thereby weakening the expression of ATP‐dependent HSPs, which significantly reversed tumor heat resistance and boosted hypothermal photothermal performance. Both in vitro and in vivo studies validated the excellent therapeutic effects of IR‐FEP‐RGD‐S‐S‐S‐Fc on 4T1 cells and 4T1 tumor‐bearing mice. Collectively, in these examples H_2_S restrains mitochondrial respiration to reverse the hypoxia condition, which amplifies oxidative stress and suppresses COX IV activity to synergize RT.

### 3.2 Controlled delivery by nanostructured peptide‐H_2_S donor conjugates

Compared with polymers, peptides own unique advantages for H_2_S delivery.^[49]^ First, peptide‐H_2_S donor conjugates are small molecules, which simplifies the preparation and purification, that is, HPLC. Unlike polymer‐based conjugates, peptide‐H_2_S donor conjugates do not have molecular weight distribution, therefore, the loading efficiency could be fixed and well controlled in each conjugate from batch to batch. Strikingly, the loading site could also be defined during the synthesis. Second, as the molecular weight of peptide‐H_2_S donor conjugates is small, the loading content of H_2_S donors could be higher than polymeric counterparts, which could somehow relieve the metabolism burden because peptides are biodegradable. More importantly, it is much facile and possible to incorporate targeting epitopes (e.g., RGD) during the synthesis of peptide‐H_2_S donor conjugates, thus facilitating the targeted delivery of H_2_S donors to the lesion site and increasing the bioavailability. However, such design has not been reported yet. As we know, the morphology of peptide‐based materials is highly dependent on peptide sequence and/or the self‐assembly pathway, which impacts the efficacy.^[50]^ Therefore, through fine‐tuning the sequence and/or controlling the self‐assembly pathway, the distribution of peptide‐H_2_S donor conjugates could be regulated in body. Thus, in this section, two parts will be discussed. The first part describes different strategies for preparing peptide‐H_2_S donor conjugates (PHDCs). The second one shows the combination of PHDCs with other modalities for cancer treatment (Table 2).

**TABLE 2:** Summary of nanostructured peptide‐H_2_S donor conjugates.

Donor	Matrix	Trigger	Morphologies	Active molecules	Reference
SATOs	Ile‐Ala‐Val‐Glu‐Glu‐Glu	Cysteine	Nanofibers	H_2_S	[51]
SATOs	Phe‐Glu‐Glu‐Glu‐Glu	Cysteine	Long twisted nanoribbons, short curved ribbon bundles	H_2_S	[52]
SATOs	Ile‐Ala‐Val‐Glu‐Glu‐Glu	Cysteine	Nanoribbon or aggregates dependent on the linker type	H_2_S	[53]
SATOs	Lys‐Glu‐Lys‐Glu Lys‐Lys‐Glu‐Glu Lys‐Glu‐Glu‐Lys	Cysteine	Nanoribbons, nanohelix	H_2_S	[26]
Phenyl thionocarbonate	Ac‐Glu‐Glu‐Phe‐Ala‐Ala‐Asn	Legumain	Aggregates	COS/H_2_S; Fe^2+^	[44]

**FIGURE 3:**
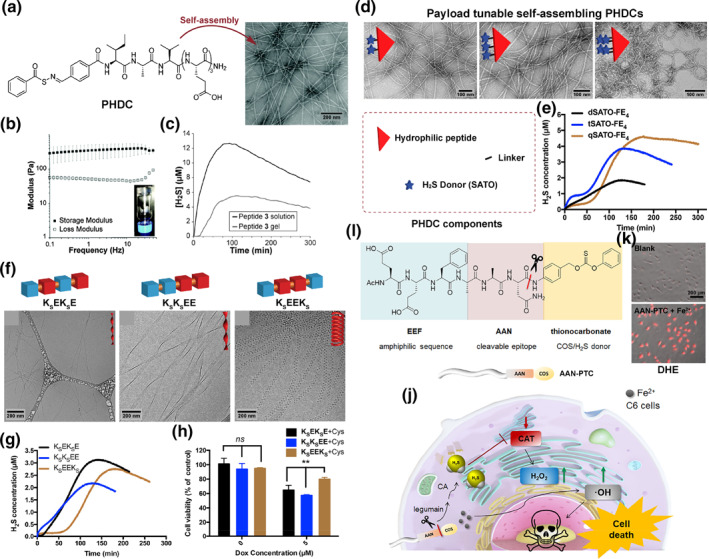
Peptide H_2_S‐donor conjugates for cancer therapy. (a) Structure and self‐assembled morphology of amphiphilic PHDCs. The PHDCs could gelate by adding Ca^2+^ (b) to regulate H_2_S release (c). Reproduced with permission.^[51]^ Copyright 2015, RSC. (d) Morphology and (e) H_2_S releasing behavior regulation by tuning SATO loading numbers. Reproduced with permission.^[52]^ Copyright 2019, ACS. Constitutionally isomeric peptides regulating the (f) morphology, (g) H_2_S releasing behavior and (h) protecting effects against DOX‐induced cytotoxicity. Reproduced with permission.^[26]^ Copyright 2018, ACS. Structure of legumain‐activated peptide COS‐donor conjugate (i), which could form complex with Fe^2+^ and achieved H_2_S‐enhance PDT (j, k). Reproduced with permission.^[44]^ Copyright 2023, Wiley.

The first example of PHDCs was reported by Matson and coworkers.^[51]^ They linked the H_2_S donor SATO to the N terminus of the aldehyde‐containing peptide Ile‐Ala‐Val‐Glu‐Glu‐Glu (Figure 3a). The obtained amphiphile could assemble into nanofilaments and further formed a self‐supporting gel upon mixing with calcium chloride (Figure 3b). They found that compared with the solution state, the H_2_S release of PHDCs from the gel state was much slower due to the slower diffusion of the trigger cysteine (Figure 3c). Then the research group extended the idea to a multiple donors‐loaded system, where two, three, or four SATOs were appended to a short peptide Phe‐Glu‐Glu‐Glu‐Glu.^[52]^ By varying the number of SATOs, the morphologies of assemblies could be tuned from long twisted nanoribbons to short curved ribbon bundles (Figure 3d). As expected, the H_2_S release behaviors varied dependent on morphologies, with assemblies formed by two SATOs liberating H_2_S faster than those of four SATOs (Figure 3e). Because it was much harder for cysteine to diffuse into the curved ribbon bundles. Remarkably, such distinct H_2_S release behaviors were also observed in cells monitored by the fluorescent probe WSP‐5. Besides, the same group also found that the release rate of H_2_S could be regulated by the linker between the peptide and the SATO.^[53]^ Collectively, these results demonstrated that a minor change in molecular design leads to remarkable variations in nanostructured morphologies, which ultimately affecting the release rate of H_2_S.

Although these examples selected above demonstrating that nanostructures were beneficial for H_2_S delivery and influenced the release rate, the relationship among the molecular design, assembled structures, and the resultant efficacy of H_2_S remains unclear. In nature, there is a type of molecules called constitutionally isomers, which are identical in chemical composition but different in connectivity of chemical bonds, usually presenting very different physical properties.[^54^, ^55^] Inspired by this, Matson and coworkers synthesized three constitutionally isomeric peptides, each of which contained two glutamic acid residues and two SATOs functionalized lysine residues [26]. Interestingly, subtle change in the position of amino acid residues led to these PHDCs self‐assembling into different morphologies in aqueous solution, two into nanoribbons of different dimensions and one into a rigid nanohelix (Figure 3f). Expectedly, the H_2_S release rates from these PHDCs highly depended on their morphologies, with the nanohelix‐forming PHDC exhibiting a complex and longest release profile (Figure 3g). Remarkably, the differences in release rate were amplified in the cell protective assay. Although all these PHDCs could mitigate the cardiotoxicity of doxorubicin (DOX) to H9C2 cardiomyocyte cells, the nanohelix‐forming PHDC exhibited more effectively than its nanoribbon‐forming constitutional isomers as well as common H_2_S donors because nanohelix‐forming PHDC prolonged the H_2_S release time (Figure 3h). This strategy highlights the interplay between structure and function from the molecular level to the nanoscale of PHDCs.

Similar to polymer‐H_2_S donor conjugates, PHDCs alone are not powerful enough to combat cancer either. Thus, they are usually integrated with other treatment modalities. Very recently, Matson and coworkers designed a legumain cleavable PHDC AAN‐PTC, which further complexed with Fe^2+^to combat glioma (Figure 3i,j).^[44]^ Once the complex (AAN‐PTC‐Fe^2+^) entered the glioma cells, legumain would cleave the Ala‐Ala‐Asn segment of AAN‐PTC to release COS, which could quickly convert into H_2_S by CA. In the presence of H_2_S, the activity of CAT was suppressed, thus ROS would accumulate into cells and induced the oxidative stress (ROS fluorescence detected by probe was enhanced almost 20 times). Meanwhile, the hydrogen peroxide (H_2_O_2_) could transform into highly cytotoxic hydroxyl radicals (•OH) by the Fe^2+^‐mediated Fenton reaction, further enhancing the oxidative stress (Figure 3k). Both effects induced C6 cells death, which was even much better than the first‐line chemotherapy drug temozolomide, that is, achieving the same inhibition rate at a much lower concentration (almost 5 times lower). Collectively, these results demonstrated that proper cancer cell‐specific delivery of H_2_S could synergize the existing therapies such as CDT to boost the therapeutic efficacy.

### 3.3 Controlled delivery by biomineralized hybrid nanoparticles

**FIGURE 4:**
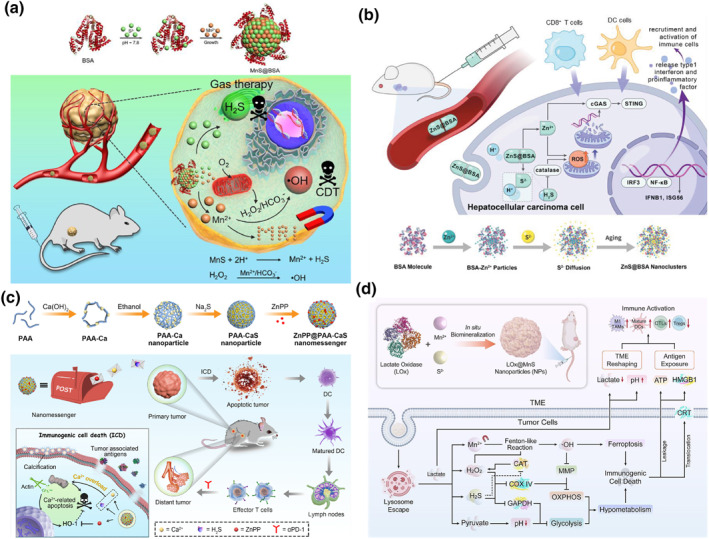
Biomineralized hybrid nanoparticles for cancer therapy. (a) Preparation of MnS@BSA nanoparticles and their synergistic GT‐CDT therapy. Reproduced with permission.^[57]^ Copyright 2020, Ivyspring. (b) ZnS@BSA nanoclusters for synergistic GT‐immune therapy. Reproduced with permission.^[59]^ Copyright 2021, Wiley. (c) Preparation of ZnPP@PAA‐CaS nanomessenger, as well as its role in Ca^2+^ overloading and ICD. Reproduced with permission.^[60]^ Copyright 2021, ACS. (d) LOx@MnS nanoparticles enabling cascaded hypometabolism and ICD. Reproduced with permission.^[61]^ Copyright 2024, Elsevier. ICD, immunogenic cell death.

Although polymer‐H_2_S donor conjugates or PHDCs could complex metal ions to fulfill the GT‐synergized therapy such as GT synergized CDT, the amount of metal ions delivered is relatively low and the complex force is not very strong, which may result in premature release during delivery. In sharp contrast, biomineralization is a powerful tactic in nature, by which biological organisms produce hierarchically structured minerals with marvelous functions.^[56]^ Inspired by this, many researchers constructed hybrid nanoparticles for H_2_S delivery and applied for H_2_S‐synergized therapy (Table 3). The pioneer work was reported by Huang and coworkers.^[57]^ Bovine serum albumin (BSA) was used as the template to absorb sulfide ions first. Then the manganese ions were added to react with sulfide and formed manganese sulfide (MnS) in situ (Figure 4a). The BSA stabilized MnS was pH‐ responsive and could release both Mn^2+^ and H_2_S gradually in acidic conditions, such as in the endosome and lysosome. The Mn^2+^ could mediate the Fenton‐like reaction to convert H_2_O_2_ into highly toxic •OH and eradicated cancer cells effectively. In vivo study further revealed that these nanoparticles could accumulate in the tumor site for a long time and restrain the growth of the tumor. Besides Mn^2+^, Fe^2+^ was also integrated into the system given that its capacity of mediating the Fenton reaction. For instance, Li and coworkers prepared amorphous ferrous sulfide nanoclusters (FeS@BSA) on BSA.^[58]^ As expectedly, the nanoclusters could degrade to liberate H_2_S gas and Fe^2+^ ions simultaneously in acidic conditions. And Fe^2+^ released from the nanoclusters converted H_2_O_2_ into the toxic ·OH effectively via the Fenton reaction. Meanwhile, H_2_S gas exerted the specific suppression effect to CAT activity of cancer cells, resulting in the accumulation of H_2_O_2_. It further facilitated the Fenton reaction of Fe^2+^ and consequently enhanced ROS stress within the cells remarkably. After intravenous administration, the nanoclusters accumulated in the tumors via the enhanced permeability and retention effect and then exerted inhibitory effect on the tumor growth. In addition, due to the presence of Fe^2+^, these nanoclusters presented strong magnetic resonance imaging (MRI) signals, thus could be used for MRI guiding therapy. Collectively, this study provided a gas‐amplified ROS‐based therapeutic platform for synergetic tumor treatment.

**TABLE 3:** Summary of biomineralized hybrid nanoparticles.

Donor	Matrix	Trigger	Models	Active molecules	Reference
MnS	BSA	Acidic pH	4T1 cancer	H_2_S, Mn^2+^	[57]
FeS	BSA	Acidic pH	Hepatocellular carcinoma (Huh7) cells	H_2_S, Fe^2+^	[58]
ZnS	BSA	Acidic pH	Human cell line (LM3), mouse HCC cell line (Hepa1‐6)	H_2_S, Zn^2+^	[59]
CaS	Poly(acrylic acid)	Acidic pH	4T1 tumor	Ca^2+^, H_2_S	[60]
MnS	LOx	Acidic pH	4T1 tumor	H_2_S, Mn^2+^	[61]
MnS	HA	Acidic pH	4T1 tumor	H_2_S, Mn^2+^, DOX	[62]

Beside CDT, immunotherapy has gained increasing attention recently because it could dynamically regulate the immune system to attack cancer cells in multiple targets and directions.[^63^, ^64^] Nevertheless, not all patients respond to this novel treatment very well. Activation of the cyclic guanosine monophosphate‐adenosine monophosphate synthase/interferon gene stimulator (cGAS/STING) signaling pathway to wake innate immunity has become a promising strategy for enhanced tumor immunotherapy.[^65^, ^66^] With this in mind, Li and coworkers prepared zinc sulfide nanoclusters in the presence of BSA (ZnS@BSA) (Figure 4b).^[59]^ Similar to FeS@BSA, ZnS@BSA decomposed to unleash zinc ions in acidic conditions and augmented cGAS/STING signals, which could promote the infiltration of CD8^+^ T cells and cross‐presentation of dendritic cells. At the same time, the released Zn^2+^ augmented ROS concentration, which was further enhanced by the H_2_S because it could weaken the ability of CAT. By these synergized effects, immunotherapy with satisfactory outcome against hepatocellular carcinoma was achieved. Similar idea was also adopted by Liu and coworkers,^[60]^ where they used poly(acrylic acid) as the template to synthesize the calcium sulfide (CaS) nanoparticles (Figure 4c). Then the heme oxygenase‐1 (HO‐1) inhibitor zinc protoporphyrin (ZnPP) was adsorbed on the particles, affording the resultant ZnPP@PAA‐CaS nanomessengers. In acidic pHs, nanomessengers underwent decomposition to unleash both Ca^2+^ and H_2_S, which synergistically elevated intracellular Ca^2+^ stress and induced subsequent cell death. Meanwhile, ZnPP acted as the messenger amplifier to suppress the antideath effect of tumor cells. Consequently, tumor cells suffered from Ca^2+^‐dependent cell death via signaling transduction cascades, which promoted to release tumor‐associated antigens and activated antitumor immunity. In vivo studies unraveled that both primary tumors and distant metastases could be markedly eradicated. More importantly, immunological memory could be endowed to arrest tumor metastasis and recurrence. This work exemplifies a chemical messenger amplified immunotherapy for enhanced cancer treatment. Although these examples exhibited promising therapeutic effects on tumor inhibition, the templates used in these systems were inert. It should be noted that examples using metal sulfide nanoparticles synthesized by the hydrothermal method rather than biomineralization for cancer treatments are not included in this review because of the different synthetic procedures.[^67^, ^68^]

To further enhance the therapeutic efficacy, functional templates were integrated. For instance, glucose oxidase (GOx) is a flavoprotein.^[69]^ In the presence of oxygen, it catalyzes the oxidation of glucose into gluconic acid and H_2_O_2_. Therefore, it has been used for the cancer starvation therapy.[^70^, ^71^] Moreover, the acid generated from the reaction triggers the degradation of pH‐responsive nanoparticles (e.g., MnS) while H_2_O_2_ provides the fuel for the Fenton or Fenton‐like reaction. However, it could not be used for intravenous injection directly because it would lower the blood glucose fast and induce the serious side effect. More recently, Huang and coworkers found that shielding the hydrophobic pocket of GOx by hydrophobic molecules would reduce the toxicity and ensure for intravenous injection.^[72]^ Similar to the parental GOx, they could be used as the template for biomineralization and treat cancers.

Lactate produced by the hyperactive glycolysis is a signaling molecule in tumor microenvironment (TME), which accelerates tumor invasion, metastasis, and immunosuppression.[^73^, ^74^, ^75^] In this regard, depletion of lactate in TME is a promising strategy to enhance the immune response and suppress tumorigenesis. The easiest way to achieve this goal is using lactate oxidase (LOx) directly, which could convert lactate into pyruvic acid and cytotoxic H_2_O_2_ in the presence of oxygen. However, direct usage of LOx faces several practical issues including easy degradation, no targeting capacity. Therefore, LOx is usually integrated with appropriate carriers to achieve on‐demand delivery.^[76]^ Inspired by the success in biomineralization of GOx, Wang and coworkers reported the preparation of LOx@MnS nanoparticles by the biomineralization for cancer treatment (Figure 4d).^[61]^ Similar to GOx, LOx could template the formation and further growth of MnS. Both in vitro and in vivo studies showed that LOx@MnS could consume lactate to reverse the immunosuppression and produce pyruvic acid as well as H_2_O_2_. The latter transformed into •OH in the presence of Mn^2+^ via the Fenton‐like reaction, which induced the apoptosis of cancer cells. Meanwhile, H_2_S released from nanoparticles combined with ROS together suppressed the tumor metabolism, which further augmented the therapeutic efficacy. Consequently, the multi‐mode effects evoked by LOx@MnS successfully elicited immunogenic cell death (ICD) and reversed high‐lactate, immunosuppressive microenvironment, suggesting the great potential for anti‐tumor immunotherapy.

Though examples discussed above find primary success in combating cancers, these nanocarriers accumulate in tumor sites mainly through passive targeting effect, that is, by enhanced permeability and retention effect. Therefore, the efficacy of accumulation is relatively low. To improve this, active targeting motifs are integrated into nanocarriers either by surface modification or using as the scaffold directly. Very recently, Wang and coworkers reported an H_2_S‐sensitized ROS bomb using hyaluronic acid (HA) as the scaffold.^[62]^ Given that HA has an intrinsic high affinity to the Cluster of Differentiation 44 (CD 44), the resultant HA@MnS nanoparticles had an active targeting capacity to CD 44 overexpressed tissues or cancer cells such as breast cancer cells. To further augment the therapeutic efficacy, an anticancer drug DOX was adsorbed on the surface, affording HA@MnS‐DOX nanoparticles. Both in vitro and in vivo studies showed that HA@MnS‐DOX nanoparticles could degrade in acidic environments to liberate Mn^2+^, H_2_S, and DOX simultaneously. DOX induced high oxidative stress to damage the DNA, which was further enhanced by the generation of ·OH via Mn^2+^ mediated Fenton‐like reaction. Meanwhile, H_2_S suppressed the activity of CAT to elevate the oxidative stress as well. These effects together caused apoptosis of cancer cells, leading to excellent anticancer outcome. The only issue should be further improved is that DOX is absorbed on the surface of nanoparticles through electrostatic interactions. The weak interaction may result in the premature release of DOX during delivery because there are so many different salts existing in the blood.

## 4 CONCLUSION AND OUTLOOKS

In summary, we have reviewed the latest advance of nanostructured H_2_S donors for cancer treatments. The examples chosen here demonstrate that these platforms hold a promising future for eradicating cancers, especially when integrated with other modalities such as CDT or immunotherapy. Upon forming nanostructures, H_2_S donors could be delivered to specific sites of interest, followed by the H_2_S release in situ to exert the therapeutic efficacy. Given that these H_2_S donors are hydrophobic, hydrophilic polymer segment, peptide sequence, and water‐soluble protein/enzyme are essential for the conjugate/nanoparticles formation. For polymer‐based strategy, PEGlyation is the most widely used hydrophilic one because of excellent biocompatibility. As such, almost all the conjugates self‐assemble into nanoparticles because of its high affinity with water. In this regard, it would not be easy to investigate the morphologic influence on therapeutic efficacy. In comparison, more choice could be chosen in peptide‐based strategy because there are several hydrophilic amino acids such as Glu, Asp, Gly, His and combination of these would generate lots of different sequences. The most striking feature is that the precise donor loading and biodegradable. It would be useful to relieve the body burden and minimize side effects after H_2_S delivery. In sharp contrast, only protein/enzymes (almost are BSA because they are cheap and stable) capable of forming spherical‐like structures in water are used for the biomineralization approach. Compared to the peptide‐based one, the biomineralization approach is more practical in terms of facile synthesis, high sulfide loading amount, better therapeutic efficacy because the dosage and the releasing rate of H_2_S from the nanoplatforms could be tuned by design, introduction of metal ions and further regulated by the internal or external stimuli. But it should be noted that long term usage of metal ions at high concentrations would induce toxicity, which should be paid attention during the treatment.

Nevertheless, hurdles still exist for wider applications or even successful translation of nanostructured H_2_S donors into clinic. First, compared with traditional modalities such as chemotherapy, the therapeutic efficacy of H_2_S‐based GT only is far from potent enough to be applied as the single mode for cancer treatment. Thus, synthesis of H_2_S donor‐based conjugates such as H_2_S donor‐chemodrug conjugates would be helpful to boost the efficacy. Besides, at present, all these donors or conjugates are synthesized in an empirical manner and then evaluated their efficacy in vitro and in vivo, which are low efficiency and time‐consuming. Given the fast development of artificial intelligence, the efficacy and safety of these molecules could be assessed, predicted, or optimized by computers. Second, the long‐term biocompatibility and clinical trials of H_2_S‐releasing nanoplatforms should be investigated systematically. At present, only short‐term evaluation has been carried out and almost show success in vitro and in vivo on mouse model. In contrast, these results may not be easily achieved on human as the mouse model or even the big animal models are significantly simpler than the realistic situation. Notably, clinical trials of H_2_S‐based GT have not been carried out but desirable. Nevertheless, high throughput screening technology would be helpful and shorten the evaluation time. Third, de novo design of disease‐inspired H_2_S donors with controlled release rate (e.g., H_2_S release could be last for 1 month) and active targeting capacity should be explored, which could reduce the side effect and off‐target toxicity.

## CONFLICT OF INTEREST STATEMENT

The authors declare no conflicts of interest.

## Data Availability

No primary research results, software or code have been included and no new data were generated or analyzed as part of this review.
